# Impact of Cuminaldehyde and Indomethacin Co-Administration on Inflammatory Responses in MIA-Induced Osteoarthritis in Rats

**DOI:** 10.3390/ph17050630

**Published:** 2024-05-14

**Authors:** Sebastião Vieira de Morais, Gustavo Pereira Calado, Rafael Cardoso Carvalho, João Batista Santos Garcia, Thyago Moreira de Queiroz, Antonio José Cantanhede Filho, Alberto Jorge Oliveira Lopes, Maria do Socorro de Sousa Cartágenes, Gerson Ricardo de Souza Domingues

**Affiliations:** 1 Biological and Health Sciences Center, Federal University of Maranhão, São Luís 65085-580, Brazil; carvalho.rafael@ufma.br (R.C.C.); jbgarcia@uol.com.br (J.B.S.G.); scartagenes@gmail.com (M.d.S.d.S.C.); 2 Programa de Pós-graduação em Ciências Farmacêuticas—PPGCF, Departamento de Farmácia, Universidade de Brasília-UnB Brasília-DF, Brasilia 70910-900, Brazil; 3 Laboratory of Nutrition, Physical Activity and Phenotypic Plasticity, Federal University of Pernambuco, Vitória de Santo Antão 55608-680, Brazil; thyago.queiroz@ufpe.br; 4 Chemistry Postgraduate Program, Federal Institute of Science Education and Technology of Maranhão, São Luís 65030-005, Brazil; 5 Bacabal Science Center (CCBa), Federal University of Maranhão, Bacabal 65700-000, Brazil; 6 School of Medicine, State University of Rio de Janeiro, Rio de Janeiro 20950-000, Brazil; gersondomingues62@gmail.com

**Keywords:** new drugs agents, osteoarthritis treatment, pharmacological interactions with herbal supplements

## Abstract

Osteoarthritis (OA) remains a chronic incurable condition, presenting substantial challenges in treatment. This study explores a novel strategy by investigating the concurrent use of cuminaldehyde, a natural compound, with indomethacin in animal models of MIA-induced OA. Our results demonstrate that the co-administration of cuminaldehyde and indomethacin does indeed produce a superior effect when compared to these compounds individually, significantly enhancing therapeutic outcomes. This effect is evidenced by a marked reduction in pro-inflammatory cytokines IL-6 and IFN-γ, alongside a significant increase in the anti-inflammatory cytokine IL-10, compared to treatments with each compound alone. Radiographic analyses further confirm the preservation of joint integrity and a reduction in osteoarthritic damage, highlighting the association’s efficacy in cartilage-reducing damage. These findings suggests that the association of cuminaldehyde and indomethacin not only slows OA progression but also offers enhanced cartilage-reducing damage and fosters the production of protective cytokines. This study underscores the potential benefits of integrating natural products with pharmaceuticals in OA management and stresses the importance of further research to fully understand the mechanisms underlying the observed potentiated effects.

## 1. Introduction

Osteoarthritis (OA) is the most prevalent form of arthritis globally, posing significant socioeconomic challenges. It arises from a complex interplay of factors including mechanical stress, genetics, metabolic components, and lifestyle factors such as physical activity and obesity, with the latter and aging identified as primary risk factors [1,2,3]. OA is characterized by cartilage degradation, synovial inflammation, and pain, which impact overall joint health and can lead to disability due to the limited healing capacity of cartilage, largely influenced by inflammatory mediators [4].

The burden of the disease is exacerbated by the aging global population, affecting individuals’ quality of life and imposing substantial healthcare costs, primarily from pain management and the necessity for joint replacement surgeries [2,5]. As the population ages, the prevalence and public health concerns of OA are expected to rise [6]. Current treatments, primarily nonsteroidal anti-inflammatory drugs (NSAIDs), offer symptomatic relief but are associated with risks such as gastrointestinal issues and cardiovascular effects, highlighting the need for careful patient monitoring [7,8]. 

The search for alternative treatments has expanded, with herbal medicines showing potential in managing OA symptoms, offering benefits such as fewer side effects and environmental sustainability. These alternatives, recognized for their anti-inflammatory and joint-protective properties, align with the trend toward greener medical solutions [9,10,11].

Our research has advanced this exploration by assessing the anti-inflammatory and antinociceptive effects of cuminaldehyde, comparing them to indomethacin, a conventional NSAID, within a sodium monoiodoacetate (MIA)-induced OA model. This comparison revealed cuminaldehyde’s comparable efficacy to indomethacin, suggesting its potential as an effective OA treatment [12]. This study aimed to evaluate whether there is a potentiated effect of cuminaldehyde when combined with indomethacin, to potentially improve the treatment outcomes of OA, emphasizing the importance of integrating traditional and novel treatments in the management of this complex disease.

## 2. Results

### 2.1. In Vivo Clinical Assays

**Figure 1:**
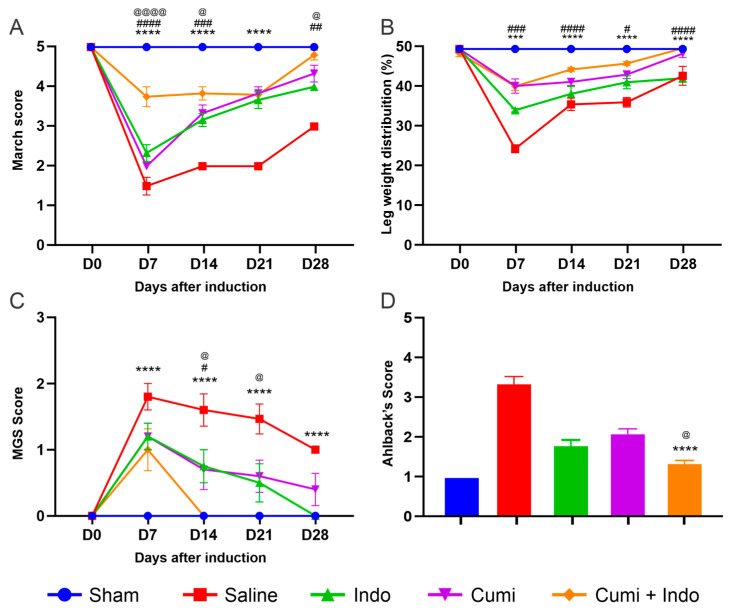
Motor activity was evaluated using the Rotarod test score (**A**), disability extent through weight distribution (**B**), spontaneous pain via the Rat Grimace Scale (**C**), and joint involvement graded by the Ahlback scoring system (**D**). Treatments included saline, indomethacin, cuminaldehyde (50 mg/kg), and an association of cuminaldehyde (50 mg/kg) + indomethacin (2.5 mg/kg), administered orally from three days post-OA induction until the study’s end, with evaluations on days 7, 14, 21, and 28. The presented data, shown as mean ± SEM, are compared against findings from the SHAM, Saline, Indomethacin, and Cuminaldehyde groups previously reported in our previous paper [12], serving to provide a comprehensive view and enhance understanding of the new treatment’s efficacy involving cuminaldehyde + indomethacin. This comparison is aimed at illustrative and comparative purposes. Statistical analysis utilized two-way ANOVA and Tukey’s test, with significance denoted by *** (*p* < 0.001), and **** (*p* < 0.0001) for the cuminaldehyde + indomethacin group versus saline, # (*p* < 0.05), ## (*p* < 0.01), ### (*p* < 0.001); #### (*p* < 0.0001) versus indomethacin alone, and @ (*p* < 0.05) and @@@@ (*p* < 0.0001) versus cuminaldehyde alone. (D = day).

The Rotarod test was used to assess forced locomotion or motor activity, indicating a uniform reduction in locomotion scores across all osteoarthritis (OA)-induced groups by Day 7, thereby confirming the successful induction of OA. The SHAM group underwent no interventions. The association therapy of cuminaldehyde and indomethacin demonstrated a statistically significant improvement in locomotion scores compared to the individual treatments with cuminaldehyde or indomethacin separately on Days 7, 14, and 28, as evidenced by gradual improvements in march parameters. As expected, throughout the study period, the combined therapy exhibited significant differences in efficacy when compared to saline-treated controls. By Day 28, the gait score of the group treated with the association was statistically equivalent to that of the SHAM (normal) group, whereas the march score of the indomethacin group remained significantly compromised (Figure 1A).

The SHAM group exhibited balanced weight distribution on hind paws at both the start and end points, indicating an absence of joint pain with an average score of 50%. The group treated with the association of cuminaldehyde and indomethacin demonstrated significant improvement compared to the group treated with either indomethacin separately or the saline group (*p* < 0.05) on all days. No significant difference was observed between the association group and the group treated with cuminaldehyde alone. By Day 28, paw weight distribution in both the cuminaldehyde and association treatment groups had normalized, whereas the group treated with indomethacin alone still showed compromised weight distribution, as depicted in Figure 1B.

Following OA induction, all groups exhibited similar increases in spontaneous pain, as measured by the Rat Grimace Scale (RGS). From Day 14, animals treated with cuminaldehyde + indomethacin demonstrated a marked and significant pain reduction (*p* < 0.001) compared to the cuminaldehyde, indomethacin, and saline-treated groups, without showing signs of pain on days 21 and 28 (Figure 1C).

Radiographic evaluations using the Ahlback Score, which measures the severity of articular damage, showed that OA induction in rats led to a significant decrease in joint space. The group treated with cuminaldehyde + indomethacin demonstrated a significantly lower Ahlback Score, indicating reduced bone loss when compared to the cuminaldehyde and negative control groups. However, there was no significant difference in the extent of bone loss reduction between the combined treatment and the individual indomethacin treatment (Figure 1D).

### 2.2. Radiological Assessment

**Figure 2:**
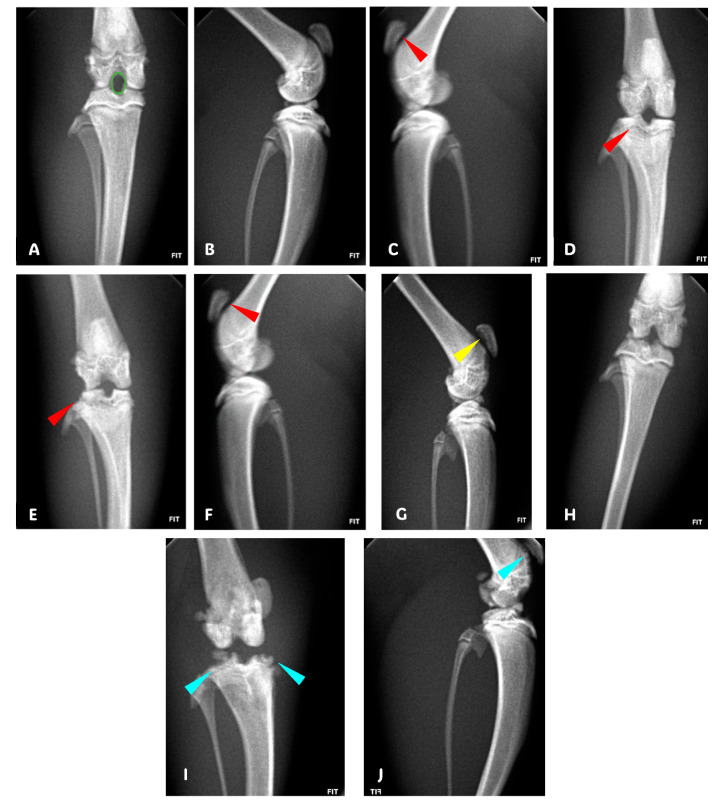
Anteroposterior (AP) and lateral radiographs demonstrating congruence between the tibial and femoral condyles and the intercondylar space in the lateral view showing intact space between the patella and femur (**A**,**B**; Sham Group); bicompartimental patellofemoral and lateral lesion [red arrowhead] (**C**,**D**; Cuminaldehyde Group); bicompartimental patellofemoral and lateral lesion [red arrowhead] (**E**,**F**; Indomethacin Group); unicompartimental patellofemoral component only lesion [yellow arrowhead] (**G**,**H**; cuminaldehyde + indomethacin Group); and dystrophy, osteopenia, and bone loss with incongruence between the sizes of the condyles and intercondylar space associated with reduction in the joint space; in the lateral view, we observe destruction of the articular cartilage, bone loss of the patella, and complete destruction of the knee joint induced with MIA with calcifications and joint subluxation [cyan arrowhead] (**I**,**J**; Saline Group).

Radiological evaluations indicated that the sham group maintained nearly perfect joint integrity, scoring between 0 and 1, reflecting intact articulating surfaces and bone structure (Figure 2A,B and Figure 1D). The separate cuminaldehyde- and indomethacin-treated groups showed mild to moderate articular changes, scoring 2.1 and 1.6, respectively, with damage primarily in the lateral and patellofemoral compartments (Figure 2C–F and Figure 1D). The combined treatment of cuminaldehyde and indomethacin resulted in mild lesions mainly in the patellofemoral compartment, scoring 1.4 (Figure 2G,H and Figure 1D). The saline-treated group exhibited severe OA features, scoring 3.4, including complete cartilage erosion, bone loss, and, in severe cases, joint subluxation (Figure 2I,J and Figure 1D).

### 2.3. Cytokine Analysis

**Figure 3:**
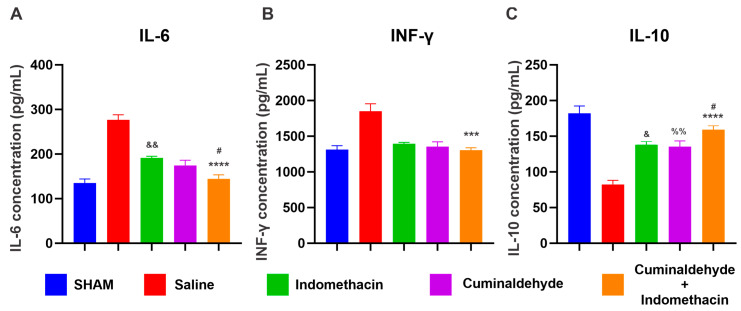
Concentration of cytokines IL-6 (**A**), INF-γ (**B**), and IL-10 (**C**) was evaluated by ELISA of the serum of the animals used in the experiments. The results are presented as means ± standard error of the mean (SEM). Data from the saline, indomethacin, and cuminaldehyde groups have been previously reported in [12]. These data are presented again solely for illustrative and comparative purposes with the new treatment combining cuminaldehyde + indomethacin, whose trial was conducted concurrently with the assays of the previous data. ***, **** Significant differences at *p* < 0.001 and 0.0001, respectively, cuminaldehyde + indomethacin compared to the saline group (CTRL−); # at *p* < 0.001 cuminaldehyde + indomethacin compared to the indomethacin group; &, && at *p* < 0.05 and 0.005, respectively, indomethacin compared to the sham group; %% at *p* < 0.005, respectively, cuminaldehyde compared to the sham group. (one-way ANOVA; Tukey).

The levels of IL-6 in the group treated with the association of cuminaldehyde and indomethacin were significantly lower than those of the groups treated with saline (*p* < 0.0001) and indomethacin (*p* < 0.05) and it did not present significant differences from the Sham group. The indomethacin group exhibited a significantly higher level of this cytokine when compared to the Sham group (*p* < 0.005). For the levels of IFN-γ, a significant difference was only detected compared to the saline group (*p* < 0.0001). The combined therapy of cuminaldehyde with indomethacin showed a level of IL-10 that did not statistically differ from the level of the SHAM group, in addition to being significantly higher than in the indomethacin group (*p* < 0.05). The levels of this cytokine were significantly lower in the indomethacin (*p* < 0.02) and cuminaldehyde (*p* < 0.008) groups (Figure 3).

## 3. Discussion

The sodium monoiodoacetate (MIA)-induced rat knee model is highly esteemed in osteoarthritis (OA) research for its ability to replicate changes in mobility, sensitivity, and joint degeneration, observable both via X-ray and microscopically in the synovial membrane. It provides insights into chondrocyte degradation, marked by the inhibition of glyceraldehyde-3-phosphate dehydrogenase, a critical marker for OA evaluation [13,14]. 

Moreover, this model is notable for simulating blood vessel formation, bone necrosis, and collapse [6], making it invaluable for testing pharmaceuticals aimed at pain relief. Its efficacy in mirroring human OA symptoms renders it an essential tool for investigating OA interventions [15,16]. Additionally, the model aids in understanding the role of neuromodulators on pain-sensing nerve endings, leading to conditions like allodynia, hyperalgesia, and increased sodium channel activity [17,18], providing a comprehensive framework for OA study.

Natural products have been extensively researched as potential novel treatments for OA, with promising results emerging from various studies [6,10,11,19]. Cuminaldehyde, a compound found in the oil of *Cuminum cyminum* L. (Apiaceae), is thought to influence pain perception through its action on the receptor ankyrin 1 (TRPA1), a protein crucial for pain signaling [20]. This underscores a significant need for more detailed investigations into its mechanisms of pain relief.

Animal-based research has confirmed the efficacy of cuminaldehyde in reducing pain, as demonstrated in tests such as the hot plate, formalin, and acetic acid-induced writhing tests. These results suggest its potential for peripheral action in pain management. The findings underscore the importance of continued research into the clinical utility of cuminaldehyde for pain treatment [21]. 

The essential oil from *Cuminum cyminum* L., with a high cuminaldehyde concentration (48%), has been shown to effectively reduce the expression levels of various mRNAs, such as inducible nitric oxide synthase (iNOS), cyclooxygenase-2 (COX-2), and the cytokines interleukin-1 (IL-1) and interleukin-6 (IL-6) in RAW 264.7 cells activated by lipopolysaccharide (LPS). It also inhibited the LPS-induced activation of nuclear factor kappa-light-chain-enhancer of activated B cells (NF-κB) and the phosphorylation of extracellular signal-regulated kinase (ERK) and c-Jun N-terminal kinase (JNK), highlighting its anti-inflammatory properties [22]. Furthermore, cuminaldehyde has been demonstrated to lower tumor necrosis factor (TNF-α) and IL-1β in rodent models, acting as an inhibitor of the 15-lipoxygenase (15-LOX) and COX-2 enzymes [12,21,23].

Studies comparing cuminaldehyde with gabapentin have found it to exhibit antihyperalgesic and antiallodynic effects similar to those of gabapentin. The analgesic effect of cuminaldehyde was significantly diminished by naloxone, suggesting its potential action on opioid receptors, particularly the µ subtype, indicating its potential as an opioid receptor agonist [12,21]. Naloxone, known for reversing opioid effects without agonistic activity, underscores the opioid-like action of cuminaldehyde in pain modulation [24].

The integration of natural products with traditional pharmaceuticals is highlighted in pharmacology and integrative medicine. This is driven by the hypothesis that combining substances could enhance their individual effects, potentially revealing synergistic, antagonistic, or neutral interactions [25,26,27,28,29,30]. Such an approach aims to improve therapeutic efficacy, possibly allowing for lower dosages and reducing adverse side effects. It shows promise for addressing drug resistance and managing pain and inflammation in chronic conditions, such as osteoarthritis [31,32,33,34,35]. In the present study, we found that combining cuminaldehyde with indomethacin produces a potentiated effect. The outcomes from animals treated with the association significantly differed from those treated with each compound separately, across all clinical trials and at various points in time evaluated. This was further corroborated by radiographic analyses.

The Ahlbäck scoring system, a radiographic evaluation tool, measures the severity of osteoarthritis (OA) in the knee by identifying changes such as joint degeneration and osteophyte formation, with higher scores indicating greater OA severity. When applied in a rat model of OA, this system tracks disease progression and evaluates the efficacy of treatments. In this study, treatment with cuminaldehyde combined with indomethacin showed significantly lower degrees of joint degeneration and bone remodeling in animals with OA, compared to those treated with indomethacin alone and the saline control, according to Ahlbäck scores.

The combination of indomethacin and cuminaldehyde is interesting from many perspectives. Although indomethacin suppresses the expression of type II collagen, predominant in cartilage, the combined treatment with cuminaldehyde likely provided the observed beneficial effects due to cuminaldehyde’s action or its potentiation. Furthermore, comparing the combined regimen with other COX-2 preferential inhibitors like meloxicam highlights that meloxicam not only improves the OARSI scores and suppresses COX-2 production but also inhibits type II collagen degradation [36,37]. The beneficial impact of the combination on joint degeneration aligns with previous findings that utilized meloxicam [38].

Cytokines play an indispensable role in modulating inflammation and are instrumental in OA pathogenesis. IL-6, known for its pro-inflammatory properties, exacerbates OA by fostering synovial inflammation, cartilage breakdown, and catabolic processes within joints. Elevated levels of IL-6 have been associated with increased pain and disease severity [39,40]. IFN-γ, linked to Th1 immune responses, possesses both pro-inflammatory and protective characteristics in OA, having the capacity to suppress inflammation and cartilage degradation, thereby offering a multifaceted strategy for managing the onset of OA [41].

Interleukin-10 (IL-10), an anti-inflammatory cytokine, plays a pivotal role in OA management by inhibiting the production of pro-inflammatory mediators and enzymes responsible for tissue and cartilage damage, underscoring its chondroprotective capabilities [40,42,43]. It modulates the immune response and curtails pro-inflammatory cytokines, thus mitigating cartilage breakdown. IL-10’s importance extends to maintaining bone and cartilage homeostasis, notably through the upregulation of osteoprotegerin (OPG) and the inhibition of the receptor activator of nuclear factor Kappa-B ligand (RANKL), preventing osteoclast maturation and reducing bone resorption [44,45]. The cytokine’s capacity to decrease IL-6 and TNF-α levels indirectly hampers osteoclastogenesis, emphasizing its therapeutic potential in OA management [46,47]. Moreover, IL-10’s inhibition of TNF-α synthesis and release safeguards osteoblasts against apoptosis, thereby supporting bone formation and integrity. It further attenuates the stimulatory impacts of TNF-α on IL-6 and matrix metalloproteinases (MMP-1 and MMP-3) production, vital for cartilage matrix degradation, thus protecting cartilage integrity [48]. By promoting chondrocyte genesis markers and hindering chondrocyte apoptosis, IL-10 is crucial in preserving cartilage structure and function, regulating inflammatory responses in chondrocytes, and diminishing the effects of inflammation induced by joint trauma.

The observed imbalance between pro-inflammatory cytokines, such as IL-6, and anti-inflammatory cytokines, like IL-10, underscores the chronic inflammation, pain, and joint deterioration characteristic of OA. The combined treatment regimen of cuminaldehyde and indomethacin effectively reduced IL-6 levels while elevating IL-10 concentrations, surpassing the efficacy of indomethacin alone. These findings suggest that the combined therapeutic approach not only impedes OA progression but also safeguards articular cartilage by fostering the production of protective cytokines. These findings are consonant with the results of a similar study utilizing meloxicam [38], wherein comparable reductions in IL-6 levels were noted. The ability of diverse treatments to modulate these cytokines may be crucial in developing more effective therapies against osteoarthritis. This dual action illustrates a promising avenue for OA treatment, emphasizing the necessity of further investigation into combined therapies that leverage both natural compounds and pharmaceuticals to ameliorate inflammation and promote joint health in OA management.

Indomethacin’s role in binding to and inhibiting phospholipase A2, as shown through X-ray crystallography and in studies involving polymorphonuclear leukocytes from rabbits and human endometrium, highlights its significance in anti-inflammatory pharmacology [49,50,51]. Furthermore, reports of indomethacin inhibiting phospholipase C [52,53,54] underscore its broad impact on inflammatory pathways.

Phospholipases, especially A2 and C, are crucial in the inflammation and pain mechanisms, initiating the release of arachidonic acid from cell membranes, a precursor for prostaglandins and leukotrienes synthesis. These enzymes are instrumental in producing inflammatory mediators and modulating immune cell activation and sensory neuron excitability [55]. The subsequent conversion of arachidonic acid via COX and LOX pathways leads to the production of prostaglandins, thromboxanes, and leukotrienes, which are key players in mediating inflammation, pain, fever, platelet aggregation, and bronchoconstriction [56,57]. This elucidates the complex interplay between phospholipases and the enzymatic pathways in propagating inflammatory and pain processes.

In light of the discussion regarding the pharmacological interaction between cuminaldehyde and indomethacin, it is pertinent to consider the implications of indomethacin’s phospholipase inhibition. Indomethacin potentially limits the availability of substrates for COX-2 inhibition through its action on phospholipase, a mechanism that could ostensibly restrict the efficacy of cuminaldehyde in inhibiting the COX and LOX pathways, which are subsequent to phospholipase activity. However, our experimental evidence has demonstrated that cuminaldehyde continues to make a significant contribution to the anti-inflammatory and antinociceptive responses, even in the presence of indomethacin. This observation suggests that cuminaldehyde exerts its therapeutic effects through multiple pharmacological mechanisms, a property that can be described as polyvalent or multi-targeted. Such a compound, by virtue of its ability to interact with several biological pathways simultaneously, offers a comprehensive approach to treatment, transcending the potential limitations imposed by the inhibition of a single enzymatic pathway. Therefore, despite the theoretical constraints posed by indomethacin’s mechanism of action, cuminaldehyde’s multi-faceted pharmacological profile enables it to maintain its efficacy, underscoring the importance of further research into compounds with multiple mechanisms of action for the management of conditions such as osteoarthritis. 

Once again, cuminaldehyde is shown to be a safe compound. During the entire 28-day duration of our study, no fatalities were observed among the animals, suggesting that extended or long-term administration of cuminaldehyde associated with indomethacin seems to be non-toxic. This aligns with earlier findings reported in the literature [12].

Future research should focus on understanding the precise mechanisms by which cuminaldehyde and indomethacin modulate inflammation and pain in osteoarthritis through studies on their interaction with cellular signaling pathways and receptors. Developing combined formulations of these compounds in optimal ratios to enhance efficacy while reducing side effects is crucial, alongside assessments of their chemical stability and bioavailability. Additionally, investigating pharmacological interactions between cuminaldehyde, indomethacin, and other osteoarthritis medications is essential. This comprehensive approach aims to deepen the knowledge of their pharmacological actions and improve their clinical application, potentially advancing osteoarthritis treatment significantly.

## 4. Materials and Methods

### 4.1. Origin of Cuminaldehyde

Cuminaldehyde was sourced from a commercial supplier (Product #135178, Sigma-Aldrich, St. Louis, MO, USA) with a certified purity of 98%. The compound was stored at ambient temperature until required for use.

### 4.2. Experimental Animals and Ethical Considerations

The study utilized 30 adult male Wistar rats (*Rattus novergicus*) from the Central Vivarium of the Federal University of Maranhão, with each of the five groups consisting of six rats. These rats, approximately 30 days old, had continuous access to standard laboratory feed and water. The environment was maintained at 23 ± 1 °C with 40–60% humidity and a 12:12 light-dark cycle. Ethical approval for the study was secured from the UFMA’s Ethics Committee in Animal Use on 3 December 2019, under protocol number 23115.031386/2019-28, adhering to the International Association for the Study of Pain’s (IASP) guidelines for animal research.

### 4.3. Experimental Design

The animals were randomized into groups of six. The SHAM group received no interventions, while the other groups were administered a sodium monoiodoacetate (MIA) injection (2 mg in 25 µL) to induce osteoarthritis in the right knee, mirroring the methodology of [12], which was conducted simultaneously, being saline solution (NaCl 0.9%) at a dose of 1 mL/kg/day (vehicle) (CTL−), Indomethacin^®^ at a dose of 2.5 mg/kg/day (CTL+), and cuminaldehyde at a dose of 50 mg/kg/day. An additional group was included, wherein participants were administered a combined treatment of Indomethacin at a dosage of 2.5 mg/kg/day + cuminaldehyde at a dosage of 50 mg/kg/day. From the third day onwards until the twenty-eighth day following the induction of osteoarthritis, the daily treatments were administered orally via gavage.

Antinociceptive effects were assessed every seven days after induction using the Weight Bearing, Rotarod, and Rat Grimace Scale tests. At the study’s end, animals were euthanized for blood collection and radiographic analysis of the affected knee, following an intraperitoneal euthanasia solution of ketamine (300 mg/kg) and xylazine (30 mg/kg).

### 4.4. In Vivo Clinical Assessments Evaluations 

#### 4.4.1. Assessment of Motor Activity Using Forced Deambulation (Rotarod Test)

During the forced ambulation evaluation, animals were placed on a rotating rod (IITC model, Life Science, Victory Blvd, Woodland Hills, CA, USA) rotating at 16 rotations per minute for 300 s. To assess the use of the affected limb, animals were observed and scored on a scale from 1 to 5, where a score of 5 represented normal paw usage, 4 indicated minor limping, 3 denoted significant limping, 2 was for intermittent non-use of the affected paw, and 1 signified total non-use of the affected paw [58].

#### 4.4.2. Incapacitation/Weight Distribution Test on Hind Paws (Weight Bearing)

To assess the use of the affected paw, animals were placed in an inclined glass enclosure with each hind paw on distinct platforms. The force exerted by each hind paw was measured in grams over five seconds, with the final value calculated as the average of three repetitions [59]. 

The formula employed for calculating weight distribution alterations is as follows:

Weight distribution (%) = APW/(APW + CPW) × 100 (1)

where APW represents the weight of the affected paw and CPW represents the weight of the contralateral paw.

#### 4.4.3. Rat Grimace Scale (RGS)

The Rat Grimace Scale (RGS) serves as a reliable method for assessing spontaneous pain in laboratory animals through the observation of changes in facial expressions [60]. The scoring system ranges from “0” indicating no pain, “1” for mild pain, to “2” for severe pain, based on adapted criteria for evaluating subjective facial pain perceptions. This involves examining alterations in features such as orbital tightness, nasal and cheek bulge, ear positioning, and whisker alterations.

All clinical assessments were conducted by three independent assessors who did not have access to each other’s evaluations.

### 4.5. Radiological Assessment

Images were acquired using a Portable Digital X-ray Unit with an image capture sensor from Diox^®^, employing the following parameters: focus-to-film distance of 10 cm, input power of 600 W, potential difference of 60 kV, and a voltage switch of 22.2V. The images were analyzed and a technical report was generated by a single specialist in imaging diagnostics. For the interpretation of the report, the modified AHLBACK [61] classification by KEYES et al. [62] was utilized. This classification is divided into five ascending degrees of severity and is based on changes observed in radiographic images of the knee in the anteroposterior (AP) and lateral (Table 1) views.

### 4.6. Assessment of Cytokine Amount

Cytokine levels (IFN-γ, IL-6, and IL-10) in the serum samples, taken from the rats at D28 time of euthanasia, were measured using the enzyme-linked immunosorbent assay (ELISA) assay. This quantification was according to the manufacturer’s protocols provided with the cytokine measurement kits and the analytical instruments used. The ELISA kits utilized for this study were acquired from R&D Systems^®^ (McKinley Place NE, Minneapolis, MN, USA).

### 4.7. Statistical Analysis

To analyze the mean differences among the experimental groups, statistical analysis was executed utilizing the Student’s *t*-test or the two-way Analysis of Variance (ANOVA) for bivariate data, with subsequent application of Tukey’s post-hoc test. The two-way ANOVA was specifically selected to assess dual sources of data variation. The threshold for statistical significance was established at a *p*-value below 0.05. The data were processed using GraphPad Prism^®^ software (version 9.0, GraphPad Software, San Diego, CA, USA).

## 5. Conclusions

In our osteoarthritis (OA) study using animal models, the combined administration of cuminaldehyde and indomethacin revealed a potentialized effect, markedly outperforming treatments with each compound alone. This result suggests effective pharmacodynamic interactions that enhance therapeutic efficacy beyond individual effects. Radiological assessments and cytokine profiles supported these findings; the association therapy notably improved joint integrity and reduced OA markers compared to controls, with significant reductions in pro-inflammatory cytokines IL-6 and IFN-γ, alongside an increase in anti-inflammatory IL-10. Future research should extend beyond this association to include other anti-inflammatory agents, aiming to fully harness cuminaldehyde’s therapeutic potential. Detailed studies on its action mechanisms, focusing on intracellular signaling, receptor interactions, and formulation optimization, are essential to amplify efficacy and reduce side effects. Moreover, understanding interactions between cuminaldehyde, indomethacin, and other OA treatments will be essential. This comprehensive approach will significantly contribute to our understanding of these compounds’ pharmacological activities and their potential integration into clinical practice for OA management.

## Figures and Tables

**Table 1:** Scoring criteria for the assessment of radiological images.

Classification	Radiological Findings
Grade 0	No Osteoarthritis: Normal radiology
Grade I	Doubtful Osteoarthritis: Questionable joint narrowing, possible marginal osteophyte
Grade II	Minimal Osteoarthritis: Possible narrowing, defined osteophyte
Grade III	Moderate Osteoarthritis: Defined narrowing, multiple osteophytes, some subchondral sclerosis, possible bone deformity
Grade IV	Severe Osteoarthritis: Marked joint narrowing, severe subchondral sclerosis, large osteophytes, defined deformity

## Data Availability

Data are contained within the article.
